# Association between body mass index and age of disease onset with clinical outcomes in paediatric-onset Crohn’s Disease (CD): a UK nation-wide analyses using the NIHR-IBD BioResource

**DOI:** 10.1038/s41430-024-01425-9

**Published:** 2024-03-12

**Authors:** Bayan Aljilani, Kostas Tsintzas, Mario Siervo, Gordon W. Moran

**Affiliations:** 1 Department of Clinical Nutrition, Faculty of Applied Medical Sciences, King Abdulaziz University, Jeddah, Saudi Arabia; 2 Translational Medical Sciences, School of Medicine, The University of Nottingham Medical School, Queen’s Medical Centre, Nottingham, UK; 3 School of Life Sciences, The University of Nottingham Medical School, Queen’s Medical Centre, Nottingham, UK; 4 National Institute of Health Research Nottingham Biomedical Research Centre, University of Nottingham and Nottingham University Hospitals, Nottingham, UK

**Keywords:** Epidemiology, Crohn's disease

## Abstract

**Background:**

The evidence on the relationship between adiposity and disease outcomes in paediatric Crohn’s disease (CD) is limited and lacks consensus.

**Aim:**

To investigate the relationship between (a) body mass index (BMI) and clinical CD outcomes (hospitalisation, surgery, disease behaviour, biologic use, extra-intestinal manifestations (EIMs)) and (b) the age of CD onset with clinical outcomes.

**Design:**

Clinical outcomes were examined in CD patients diagnosed at age <17 years and enroled in the National Institute for Health Research IBD-UK BioResource at a median age of 24 years. All outcomes and BMI were recorded at the time of enrolment. Participants were categorised into normal (<25 kg/m^2^) and high (≥25 kg/m^2^) BMI. Age at disease diagnosis was categorised into pre-puberty/early puberty (<11 years), puberty (11–14 years) and post-puberty (15–17 years). Spearman rank correlation was used to test the associations between continuous variables and chi-square test to compare categorical variables.

**Results:**

848 participants with CD were included (51.8% males) and median age at diagnosis was 14 years. Participants with high BMI experienced a greater frequency of EIMs (*P* = 0.05) than those with low BMI (1 type of EIM: 18.5% vs. 13.2%, respectively; ≥2 types of EIMs: 7.8% vs. 5.6%, respectively). Age at diagnosis and BMI showed weak correlations with corticosteroid use (*ρ* = 0.08, *P* = 0.03 and *ρ* = −0.09, *P* = 0.01; respectively). An early diagnosis (<11 years) was associated with higher occurrence of stenosing and penetrating disease behaviour (*P* = 0.01) and hospitalisations (*P* < 0.001).

**Conclusions:**

A higher BMI and an earlier age of disease onset are associated with worse CD clinical presentation.

## Introduction

Inflammatory bowel disease (IBD) is a chronic, relapsing and remitting inflammatory condition affecting the gastrointestinal tract and has significant health implications [1]. IBD is predominantly sub-divided into Crohn’s disease (CD) and ulcerative colitis (UC), which are characterised by diverse epidemiological, pathogenic and clinical characteristics [2]. The incidence rates of IBD have increased rapidly in developed countries, predominantly in northern Europe, the United Kingdom (UK), and North America [3]. Specifically in the paediatric population, the incidence rate of inflammatory bowel disease is 10.54/100,000/year and is higher in males (10.84/100,000/year) compared with females (6.69/100,000/year) [4]. Although the peak incidence of CD occurs in young adulthood, 25% of disease onset occurs during childhood [5]. Adiposity rates have also increased significantly in the developed countries, especially in the UK [6, 7]. Nearly 26% of children in England aged 2–15 years are overweight or obese [8]. Globally, around 39 million children under the age of 5 have been reported as overweight or obese in 2020 [9]. The incidence and prevalence of IBD is also raising worldwide in parallel with the obesity epidemic [10].

Historically, an underweight or malnourished nutritional status was frequently linked to paediatric IBD. However, recent observations suggest a paradigm shift with more overweight rather than underweight children presenting with IBD [11]. Indeed, an overweight or obese status is observed in nearly one in five children with CD [12]. The association between obesity and chronic inflammation has been extensively studied in non-IBD populations [13, 14]. Obesity is associated with a low grade inflammatory state [15, 16], which may be triggered by adipocyte hypertrophy and consequent secretion of pro-inflammatory markers, including interleukin (IL)-6, IL-8, IL-1β, C-reactive protein (CRP) [17], tumour necrosis factor-α (TNF-α) [18] and monocyte chemoattractant factor [19]. Obesity related metabolic disorders are linked to higher volumes of visceral adipose tissue (VAT) [20]. This increase in VAT is commonly observed in both adult and paediatric patients with CD and has been studied as a potential factor contributing to the development and progression of the disease [21].

Although obesity has been linked to more severe clinical outcomes in adult patients with CD [22–24], some studies showed no or weak relationship between obesity and adverse clinical outcomes [25, 26]. The existing literature on the association between obesity and adverse clinical outcomes in paediatric-onset populations is also limited and conflicting [12, 27–31]. Most studies included small cohorts [28–30, 32], used subjective outcomes to assess disease activity [31], have been undertaken in an inpatient setting where any association between obesity and clinical outcomes may be falsely amplified [27], or used alternative definitions of obesity [33]. In the present study, the NIHR IBD BioResource was explored to undertake a UK-wide analysis to understand the relationship between a high BMI and CD outcomes as the primary study outcome. We used standard BMI-based definitions of obesity in a large cohort of patients with CD and examined objective disease outcomes to investigate this relationship. Moreover, we interrogated this relationship in a cohort with paediatric onset of CD and used a long-term follow-up period to allow sufficient disease duration to better observe the interaction between BMI and disease outcomes. Additionally, the association between clinical outcomes and the age of disease onset was explored as a secondary outcome.

## Methods

### Characterisation of the cohort

Data were obtained from the National Institute for Health Research (NIHR) IBD BioResource- launched on 4th April 2016 as part of the UK BioResource that encompasses a large cohort of ‘recallable’ CD patients on whom clinical details were ascertained at enrolment [34]. We conducted a retrospective study to assess the relationship between BMI and clinical outcomes in CD. BMI was used to divide the patient population into cohorts of normal weight (<25 kg/m^2^) and overweight and obesity (≥25 kg/m^2^). The population was also stratified into three age groups to consider differences in sexual maturation and included: (A1 group) pre-puberty/early puberty (<11 years), (A2 group) puberty (11–14 years) and (A3 group) post-puberty (15–17 years).

All the participants signed an informed consent. Patients were excluded if they were ≥18 years at age of diagnosis or had missing data for key exposure and outcome variables. Those with UC or IBD-unclassified (IBDU) were also excluded. Patients aged ≤17 years at diagnosis of CD and who were between the age of 16 to 30 years at consent were included in the analysis. CD phenotype data, including disease duration and behaviour, hospitalisation, frequency of drug therapies, frequency of surgeries and extra-intestinal manifestations (EIMs), were ascertained at NIHR IBD BioResource enrolment.

### Characterisation of the outcome variables

The key outcomes of interest were (a) hospitalisation, (b) surgery and evidence of disease progression, specifically disease behaviour, (c) usage of therapeutic drugs, and (d) presence of EIMs. Hospitalisation was defined as Yes or No with unknown assumed to be negative for a hospitalisation history. Disease behaviour was stratified into B1-inflammatory, B2-stenosing and B3-internal penetrating according to disease behaviour in Montreal classification [35]. The surgical resections included colectomy and ileostomy, colectomy and ileo-anal pouch, defunctioning ileostomy, colostomy, drainage of intra-abdominal abscess, ileal or jejunal resection, ileal or jejunal stricturoplasty, ileocaecal resection, right hemicolectomy, partial colectomy, proctectomy, stricturoplasty, insertion of seton suture, drainage of perianal abscess, perianal fistula repair, closure of stoma and other. Drug therapies were classified into 4 groups: immunosuppressants, biological drugs, corticosteroids, and 5-aminosalicylates. Immunosuppressants included thiopurines (azathioprine or mercaptopurine), methotrexate or ciclosporin. Biological drugs included infliximab, adalimumab, vedolizumab and ustekinumab. EIMs included primary sclerosing cholangitis, enteropathic arthritis, erythema nodosum, iritis/uveitis, orofacial granulomatosis, psoriasis and ankylosing spondylitis. Details relevant to EIMs and surgeries are presented in Table 1 of the Online Supplementary Material.

**Table 1:** Descriptive characteristics of the whole study population and stratified by sex.

	All Participants	Males	Females
N	848	439	409
Age at consent (years)	24 (20, 27)	24 (20, 26)	24 (20, 27)
Age at diagnosis (years)	14 (11, 16)	14 (12, 16)	14 (11, 16)
BMI (kg/m^2^)	22.6 (20.4, 25.6)	22.3 (20.2, 25.2)	22.8 (20.5, 26.1)
BMI groups			
<25 kg/m^2^	605	325	280
≥25 kg/m^2^	243	114	129
Ethnicity			
Not stated	34	19	15
White or British White	753	380	373
Asian or Asian British	31	19	12
Black or Black British	8	7	1
Mixed	17	10	7
Other ethnic	5	4	1
Smoke			
None smoker	656	335	321
Past smoker	104	54	50
Current smoker	88	50	38
Alcohol intake			
No	274	141	133
Yes	574	298	276
Diet preference			
None	715	387	328
Vegetarian	28	7	21
Pescatarian	18	4	14
Vegan	9	3	6
Others	78	38	40
Disease duration (years)	10 (6, 14)	10 (6, 13)	10 (7, 14)
Disease behaviour			
B1-inflammatory	595	318	277
B2-stenosing	169	84	85
B3-internal penetrating	84	37	47
Hospatilisation			
No	321	159	162
Yes	527	280	247
Total current Immunosuppressant agent usage			
0	444	220	224
1	401	217	184
2	3	2	1
Total current biological agent usage			
0	348	168	180
1	500	271	229
Current corticosteroid usage			
No	792	407	385
Yes	56	32	24
Total current mesalazine usage			
No	757	391	366
Yes	91	48	43
Total surgeries			
No history of surgery	518	262	256
History of 1 resection	209	107	102
History of 2 or more resections	121	70	51
Total EIMs			
No EIMs	670	366	304
1 EIM	125	57	68
≥2 EIMs	53	16	37

Continuous variables were described as median (inter-quartile range). Categorical variables were described by reporting absolute frequency.

*N* number of cases, *BMI* body mass index, *EIMs* extra-intestinal manifestations.

### Statistical analysis

Continuous variables were presented as medians (interquartile range) and categorical variables as frequencies and percentages. Chi-square test was used to compare categorical variables. Spearman rank correlation was used to test the associations between continuous variables. Significance was defined as *P* ≤ 0.05 (two-tailed). Analyses were carried out using Microsoft Excel Worksheet for Windows (Version 15.0, Microsoft Corp., Redmond, WA, SA) and IBM SPSS for Windows (Version 28.0, IBM Corp., Armonk, NY, USA).

## Results

### Cohort demographics

**Fig. 1:**
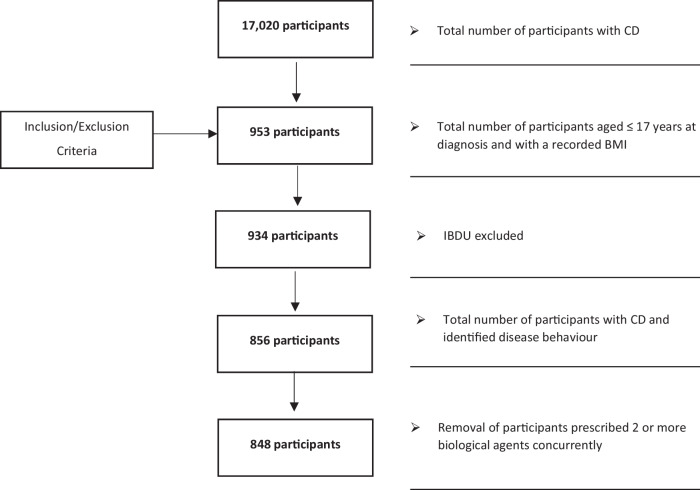
Consort diagram. Flow chart describing the selection of the final population sample included in the analysis. CD Crohn’s disease, BMI body mass index, IBDU Inflammatory bowel disease unclassified.

At data lock, on 11th March 2022, 17,020 patients were enroled (Fig. 1). After applying the inclusion and exclusion criteria described in Methods, 16,067 participants were removed. 953 participants were included with an age <18 years at IBD diagnosis and with a reported BMI. Additionally, 19 participants were removed due to having a diagnosis of IBD unclassified and 78 participants because they had an unidentified disease behaviour. Eight participants were co-prescribed 2 or more biological agents concurrently and hence were removed from the cohort as it was felt these may be data entry errors and not representative of typical paediatric CD patients in the UK.

A total of 848 participants were included in the final analysis and their characteristics are displayed in Table 1. The cohort had a similar gender split (51.8% males and 48.2% females) and were of a predominant white or British white (88.8%) demographic, with a median age at diagnosis of 14 years (range 11–16 years) and a median age at consent and inclusion in the NIHR BioResource of 24 years (range 20–27 years). The median BMI at age of consent was 22.6 kg/m^2^ (range 20.4–25.6 kg/m^2^). The majority of the participants (*N* = 605) had a BMI < 25 kg/m^2^ with 243 participants (29%) having a BMI ≥25 kg/m^2^ at inclusion in the NIHR IBD BioResource.

### Clinical characteristics of the cohort

The majority (70.2%) of patients had an inflammatory disease behaviour, with the rest having stenosing (19.9%) or penetrating (9.9%) disease behaviour. Almost half of the cohort had been exposed to one immunosuppressant (47.3%) or biological agent (59.0%), whereas the majority had not been exposed to 5-aminosalicylates (89.3%) or corticosteroids (93.4%). Most of the participants were naïve to surgery (61.1%) and never experienced EIMs (79.0%) (Table 1).

### Outcome analyses

No significant difference in the key outcomes was observed between patients with a low or high BMI except in the prevalence of EIMs. There was no difference in hospitalisation, surgery, disease behaviour and medication usage between patients with a low or high BMI (Table 2). More participants with high BMI experienced 1 EIM than those with a low BMI (BMI ≥ 25 = 18.5% vs. BMI < 25 = 13.2%, *P* = 0.05). Presence of ≥2 EIMs was also more common in patients with a higher BMI (BMI ≥ 25 = 7.8% vs. BMI < 25 = 5.6%, *P* = 0.05). There was also a trend for higher use of corticosteroids in the lower BMI group (BMI < 25 = 7.6% vs. BMI≥25 = 4.1%; *P* = 0.06).

**Table 2:** Clinical outcomes in patients with Crohn’s disease stratified by BMI and age at diagnosis.

	BMI < 25	BMI ≥25	*P* values	Age at diagnosis < 11 years A1 group	Age at diagnosis 11–14 years A2 group	Age at diagnosis 15–17 years A3 group	*P* values
Age at diagnosis	(*N* = 605)	(*N* = 243)	0.7	—	—	—	—
<11 years	19.1%	16.9%					
11–14 years	38.8%	40.7%					
15–17 years	42.1%	42.4%					
BMI	—	—	—	(*N* = 156)	(*N* = 334)	(*N* = 358)	0.7
<25				73.7%	70.4%	71.2%	
≥25				26.3%	29.6%	28.8%	
Disease behaviour	(*N* = 605)	(*N* = 243)		(*N* = 156)	(*N* = 334)	(*N* = 358)	
B1-inflammatory	71.3%	67.5%	0.3	59.0%	71.6%	73.8%	0.01
B2-stenosing	19.8%	20.2%		25.6%	20.4%	17.0%	
B3-internal penetrating	8.9%	12.3%		15.4%	8.0%	9.2%	
Hospitalisation	(*N* = 605)	(*N* = 243)		(*N* = 156)	(*N* = 334)	(*N* = 358)	
No	38.2%	37%	0.8	23.7%	39.2%	42.7%	<0.001
Yes	61.8%	63%		76.3%	60.8%	57.3%	
Immunosuppressants	(*N* = 605)	(*N* = 243)		(*N* = 156)	(*N* = 334)	(*N* = 362)	
0	51.9%	53.5%	0.9	51.92%	47.9%	56.6%	0.2
1	47.8%	46.1%		47.44%	51.8%	43.1%	
≥2	0.3%	0.4%		0.64	0.3%	0.3%	
Biological drug	(*N* = 605)	(*N* = 243)		(*N* = 156)	(*N* = 334)	(*N* = 358)	
0	39.7%	44.4%	0.2	39.7%	41.3%	41.3%	0.9
1	60.3%	55.6%		60.3%	58.7%	58.7%	
Corticosteroids	(*N* = 605)	(*N* = 246)		(*N* = 159)	(*N* = 156)	(*N* = 358)	
0	92.4%	95.9%	0.06	96.9%	96.8%	91.3%	0.06
1	7.6%	4.1%		3.1%	3.2%	8.7%	
Mesalazine	(*N* = 605)	(*N* = 243)		(*N* = 156)	(*N* = 334)	(*N* = 358)	
0	89.6%	88.5%	0.6	86.5%	90.4%	89.4%	0.4
1	10.4%	11.5%		13.5%	9.6%	10.6%	
Total surgeries	(*N* = 596)	(*N* = 243)		(*N* = 156)	(*N* = 334)	(*N* = 358)	
0	61.6%	58.4%	0.2	52.6%	62.6%	63.4%	0.2
1	23.3%	28.8%		28.8%	23.4%	24%	
≥2	15.1%	12.8%		18.6%	14%	12.6%	
Total EIMs	(*N* = 605)	(*N* = 243)		(*N* = 156)	(*N* = 334)	(*N* = 358)	
0	81.2%	73.7%	0.05	73.7%	7 7.5%	82.7%	0.1
1	13.2%	18.5%		18.6%	16.5%	11.5%	
≥2	5.6%	7.8%		7.7%	6.0%	5.8%	

Categorical variables were described by reporting percentage frequency. Chi-Square Test was used to evaluate between-group differences for categorical variables.

*N* number of cases in each group, *BMI* body mass index, *EIMs* extra-intestinal manifestations.

Earlier age at diagnosis was associated with significantly (*P* = 0.01) higher rate of stenosing (25.6%) and penetrating (15.4%) disease behaviour in the A1 (<11 years) group compared with both the A2 (11–14 years) group (stenosing: 20.4% and penetrating: 8.0%) and A3 (15–17 years) group (stenosing: 17.0% and penetrating: 9.2%). Participants with an early age at diagnosis (<11 years) experienced significantly more hospitalisations (76.3%) than those diagnosed at a later age (15–17 years = 57.3%; *P* < 0.001). No significant difference was found in medication usage, prevalence of EIMs and surgeries across age groups. Interestingly, the use of corticosteroids tended to be higher (*P* = 0.06) in those diagnosed at a later age (15–17 years = 8.7%) when compared with those diagnosed at an earlier age (<11 years = 3.1% and 11–14 years = 3.2%) (Table 2). A weak negative correlation was observed between BMI and corticosteroid use (*ρ* = −0.09, *P* = 0.01), whereas a weak positive correlation was observed between age at diagnosis and corticosterids use (*ρ* = 0.08, *P* = 0.03) (Table 3).

**Table 3:** Associations between BMI, age of diagnosis and clinical outcomes in the whole population of patients with Crohn’s Disease.

	BMI (kg/m^2^)	Age at diagnosis (years)
Age at diagnosis (years)	*ρ* = 0.003	—
	*P* = 0.92	
BMI (kg/m^2^)	—	*ρ* = 0.003
		*P* = 0.92
Immunosuppressants	*ρ* = −0.05	*ρ* = −0.05
	*P* = 0.12	*P* = 0.13
Biological drugs	*ρ* = −0.05	*ρ* = −0.003
	*P* = 0.16	*P* = 0.93
Corticosteroids	ρ = −0.09	*ρ* = 0.08
	*P* = 0.0**1**	*P* = 0.03
Mesalazine	*ρ* = 0.004	*ρ* = −0.018
	*P* = 0.91	*P* = 0.61
Total surgeries	*ρ* = 0.01	*ρ* = −0.06
	*P* = 0.85	*P* = 0.06
Total EIMs	*ρ* = 0.06	*ρ* = −0.06
	*P* = 0.10	*P* = 0.09

Number of participants = 848. Spearman rank correlation was used to test the associations between variables.

*BMI* body mass index, *EIMs* extra-intestinal manifestations.

## Discussion

This study investigated whether a higher BMI was related to worse outcomes in paediatric-onset CD based on measures of hospitalisation, surgery, disease behaviour, biologic use and the frequency of EIMs. It also explored the association between age of disease onset and these clinical outcomes. The findings revealed that patients with higher BMI experienced more EIMs and a negative correlation between BMI and corticosteroid usage was observed. This may reflect the negative effect of disease activity and hence corticosteroid use on body weight, with anorexia and sarcopenia (reduced muscle mass) being typical signs of chronic disease activity. It was also shown that CD patients diagnosed in the pre-pubertal stage (<11 years) experienced a high prevalence of stenosing and penetrating disease and hospitalisations. A younger age at diagnosis was also positively correlated with corticosteroid use.

The effects of excess adiposity on disease severity and development of disease complications in paediatric CD is still unclear. Previous studies reported no correlation between the obesogenic state and disease severity [12, 27–29], surgery [12, 27, 29, 30] or hospitalisation rates [27, 29, 36] among paediatric IBD patients. However, a positive association between a high BMI and adverse IBD outcomes has been observed in some studies showing an increase in surgical risk [36] and rate of disease exacerbation [28, 30, 31], therapy failure [28] and hospitalisation [28, 30] in paediatric IBD populations with a high BMI. However, it must be noted that most of those studies [28, 30, 31] defined high BMI by ≥85th percentile except for Von Graffenried et al. [36], who defined high BMI by ≥90th percentile.

In the adult IBD literature, some studies have shown the incidence of hospitalisation [22], and CD-related surgery [23] is significantly higher in patients with a high BMI. However, the inverse has also been reported with a decrease in surgical and hospitalisation and biologic usage observed in patients with a high BMI [26]. Results from the Swiss Inflammatory Bowel Disease cohort using a multivariate regression model based on datasets from 3075 patients, showed that obesity was negatively associated with disease remission in CD (odds ratio 0.61, 95% confidence interval 0.40–0.92, *P* = 0.02), but not UC [37]. Another study by the same group in a paediatric population showed no relationship between obesity and disease progression as defined by a clinically complicated disease phenotype [36]. In contrast, prior IBD-related surgery was associated with overweight and obesity in a cohort of 1598 children with CD [14]. However, the present study found no association between obesity and a complicated disease behaviour. It is important to note that previous studies defined obesity based on the World Health Organisation (WHO) child growth chart standards [33], or defined their at-risk population as one with a BMI ≥85th percentile [14] or BMI > 30 kg/m^2^ [36], while we employed a more conservative approach of defining our at-risk population as one with a BMI of >25 kg/m^2^.

Concomitant medication usage is a surrogate of disease severity that may be utilised along with hospitalisation and surgery. We observed no association between 5-aminosalicylic acid, immunosuppressants, biologic use and BMI. Similarly, no association between BMI and 5-aminosalicylic acid [31], immunosuppressants [12, 28, 31] and biologic [12, 29, 31, 32, 36, 38, 39] use has been observed in the literature with only a single, albeit small study showing an association between a high BMI (>75th percentile) and both disease exacerbation and the need of biologic therapy [28]. The results from the present study indicated no positive association between high BMI and corticosteroids use which is in accordance with the literature [12, 28, 29, 31]. Interestingly, we observed a weak negative correlation between BMI and corticosteroid usage probably indicating that a cohort with more active disease used corticosteroids more often, with chronic active disease being inversely related to body weight due to a change in eating behaviour [40], sarcopenia and protein-losing enteropathy. However, weight gain and fluid retention are the most common reported adverse outcomes of long-term use of steroids [41, 42], though this treatment strategy is rarely used [43] and goes against published guidelines [44]. Interestingly, a positive correlation was observed between corticosteroid use and age at diagnosis. This probably reflects the practice of using enteral nutrition to induce remission in younger cohorts and corticosteroids in older cohorts [45].

In the present study, young Crohn’s disease patients with a high BMI also experienced significantly more EIMs with this observation holding true even in patients experiencing two or more of these manifestations. This may corroborate our hypothesis that the obesogenic state is pro-inflammatory as the incidence of some extra-intestinal manifestations such as arthritis and aphthous ulcerations are intimately linked with intestinal inflammatory activity [36, 46].

CD patients diagnosed in a pre-pubertal age (<11 yrs), experienced a higher incidence of stricturing disease and hospitalisation when compared to an older age at diagnosis. These findings reflect previous observations showing that in paediatric CD, a stenosing or penetrating disease behaviour doubled during the follow-up period from 29% at diagnosis to 59%, reaching a relative plateau after 9–10 years of follow-up [47]. Our results are consistent with Polito et al. [48] in which an earlier age of diagnosis correlated with severe disease behaviour, although they compared an age group that was <20 years old with an older group that was >40 years old.

The present study has some limitations. Although the CD patients were included in this study based on their age at diagnosis (≤17 years), the relevant data sets of BMI and clinical outcomes were recorded at the age of consent to their inclusion in the BioResource database. Due to the cross-sectional nature of this database, BMI at diagnosis and its change over time until enrolment in the database was not available. Nevertheless, the approach in the present study allowed us to interrogate the association between BMI and objective disease outcomes in patients with paediatric-onset disease over a considerable follow-up period until early adulthood. The Tanner staging was not addressed, and therefore the pubertal and pre- or post-pubertal stages were based solely on the age of the child. Finally, the definition of a high BMI in this study was >25 kg/m^2^. A more at-risk population would have been one with a higher BMI of >30 kg/m^2^, but a lack of patients within that range in the database precluded this sub-analysis.

## Conclusions

Our findings have shown that both a high BMI and an early age of disease onset worsen disease outcomes, with a high BMI being linked with higher presence of EIMs and the early age at diagnosis being linked with higher prevalence of stricturing disease behaviour and hospitalisation. Our results do not impute causality but are hypothesis-generating and warrant further investigation. Future prospective cohort studies in paediatric patients with CD are required to investigate the causal link between adiposity and CD clinical outcomes to improve the prediction of disease severity, response to therapy and corticosteroids dependency, hospitalisation rate, risk of EIMs and need for surgery.

### Supplementary information

Supplementary Table 1

## Data Availability

Data generated or analysed during this study are included in this published article and its supplementary table. Additional data are available from the corresponding author upon reasonable request.
