# Supplementary material for: Association between body mass index and age of disease onset with clinical outcomes in paediatric-onset Crohn’s Disease (CD): a UK nation-wide analyses using the NIHR-IBD BioResource
Source: Eur J Clin Nutr. 2024 Mar 12;78(6):534–40. doi: 10.1038/s41430-024-01425-9 (PMC11182742; doi:10.1038/s41430-024-01425-9)
Supplement: Supplementary file 1 — Supplementary Table 1 [file 41430_2024_1425_MOESM1_ESM.docx]

**Supplementary material**

| **Supplementary Table 1. Descriptive characteristics of immunosuppressants, biological exposure and total comorbidities in the whole study population (n= 848)** | |
| --- | --- |
| Immunosuppressants (N)  Thiopurine  Methotrexate  Ciclosporin | 368  37  2 |
| Biological drug (N)  Infliximab  Adalimumab  Vedolizumab  Ustekinumab | 244  167  41  48 |
| Corticosteroids (N)  Oral steroids (prednisolone or budesonide)  IV steroids | 56  0 |
| Mesalazine (N) | 91 |
| Total surgeries (N)  Colectomy and ileostomy  Colectomy and ileo-anal pouch  Defunctioning ileostomy colostomy  Drainage of intra-abdominal abscess  Ileal jejunal resection  Ileal jejunal stricturoplasty  Ileocaecal resection right hemicolectomy  Partial_colectomy  Proctectomy  Stricturoplasty  Insertion of seton suture  Drainage of perianal abscess  Perianal fistula repair  Closure of stoma  Other | 57  6  35  7  59  8  127  22  9  15  52  59  23  5  9 |
| EMIs (N)  Primary Sclerosing Cholangitis  Enteropathic arthritis  Erythema Nodosum  Iritis / Uveitis  Orofacial Granulomatosis (Oral Crohn's)  Psoriasis  Ankylosing Spondylitis | 9  50  34  13  37  48  7 |

N, number of cases, EIM, extra-intistinal manifestations
